# Real-world impact of point-of-care testing for SARS-CoV-2 in an ambulatory setting of an integrated health network

**DOI:** 10.1017/ash.2026.10362

**Published:** 2026-04-24

**Authors:** Robert J. Williams, Li Dong, Josh Van Otterloo, Nancy Grisel, Payal Patel, Bert K. Lopansri

**Affiliations:** 1 Department of Internal Medicine, University of Utah, Salt Lake City, UT, USA; 2 Institute of Global Health and Infectious Diseases, University of North Carolina, Chapel Hill, NC, USA; 3 Division of Infectious Diseases and Clinical Epidemiology, Intermountain Health, Murray, UT, USA; 4 Central Laboratory, Intermountain Health, Murray, UT, USA

## Abstract

**Objective:**

Point-of-care testing (POC) is an important tool for diagnosing SARS-CoV-2. This objective of this study was to evaluate the real-world performance of rapid molecular (rPCR) and rapid antigen (rAg) methods and their impact on antibiotic prescribing.

**Methods:**

We retrospectively analyzed adult patients tested for SARS-CoV-2 at outpatient clinics within an integrated health network from December 2021 to March 2023 to determine antibiotic use in settings where POC testing for SARS-CoV-2 were deployed. Patients were included if their initial test was with rPCR or rAg. We conducted a 3:1 propensity score matching analysis to compare rPCR and rAg testing outcomes. Univariate and multivariate logistic regression analyses were used to identify predictors of antibiotic use within 24 hours of a positive test.

**Results:**

Of 104,364 patients that underwent testing with a rapid test in the ambulatory setting, 24,133 (29.0%) tested positive for SARS-CoV-2. Molecular testing had the highest percent positive compared to antigen testing (26.2% vs 20.8%). Overall, antibiotics were prescribed to 10% of positive cases, with higher rates following rAg testing (13% vs 10%, P < .001). Chronic lung disease (OR: 1.4 [1.2–1.7], P < .001) and white non-Hispanic race (OR: 1.5 [1.1–2.1], P = .014) were associated with an increased odds of receiving antibiotics while a rPCR test (OR: 0.8 [0.7–1.0], P = .03) was associated with a significantly decreased odds or receiving antibiotics.

**Conclusions:**

POC molecular testing outperformed antigen testing in SARS-CoV-2 detection and was associated with lower antibiotic prescribing, supporting its role in antimicrobial stewardship.

## Introduction

The Coronavirus disease 2019 (COVID-19) pandemic has undermined antimicrobial stewardship efforts worldwide. Multiple studies have highlighted the extensive use of broad-spectrum antibiotics for patients with COVID-19 in the inpatient setting despite low rates of confirmed bacterial coinfection.^1^ Less is known regarding rates of antibiotic use for patients with confirmed COVID-19 in the ambulatory setting. Two small, single-center studies from early in the pandemic reported low rates of outpatient antibiotic prescribing,^2,3^ however, a study of over 1 million adults age 65 and older showed that nearly 30% of COVID-19 outpatient visits were associated with an antibiotic prescription.^4^

Early in the pandemic, diagnostic testing for SARS-CoV-2 was challenging due to reagent and testing shortages. Confirming a diagnosis relied on high-throughput, lab-based testing with longer turnaround times. Point of care testing to guide the clinical decision-making process was not widely available. As supplies became more abundant, use of rapid, point-of-care (POC) testing with nucleic acid amplification tests and antigen tests were adopted in the ambulatory setting with the potential to improve antimicrobial utilization. While both testing modalities have a high specificity of around 0.98–0.99, compared to rapid antigen tests, rapid nucleic acid amplification tests tend to have better sensitivities (0.93 vs 0.75)^5^; the impact of these performance differences on antimicrobial prescribing practices remains poorly understood. Exploring the role of rapid, POC diagnostics in outpatient settings could offer valuable insights into optimizing antimicrobial usage for confirmed COVID-19 cases. As the COVID-19 pandemic continues to evolve, it is important to understand the impact of rapid testing on antimicrobial use in patients diagnosed with COVID-19 to improve ambulatory antibiotic stewardship and mitigate unintended consequences such as adverse effects to antibiotics.

The objectives of our study were to describe the real-world performance of rapid, POC testing with rapid PCR (rPCR) compared to POC rapid antigen (rAg) testing and to determine the impact of POC SARS-CoV-2 testing on antibiotic use and COVID-19 antiviral use in the ambulatory setting.

## Materials and methods

### Setting

Intermountain Health is an integrated healthcare network, which during the study period consisted of 26 hospitals, 25 urgent care clinics, and 385 primary care clinics. During the study period, ambulatory SARS-CoV-2 testing was with rPCR (SARS-CoV-2, Influenza A and B, RSV; Cepheid) in all Intermountain urgent care clinics, rAg (SARS-CoV-2 and Influenza A and B by antigen test, BD Veritor) in primary care clinics, and SARS-CoV-2 RT-PCR (ThermoFisher, Scientific) in a centralized lab for Telehealth and all other ambulatory testing. We collected data from all patients who underwent POC testing for SARS-CoV-2 in urgent care and primary care clinics from December 1, 2021 to March 31, 2023. Point of care testing was conducted using emergency use authorized reagents in accordance with the manufacturer’s recommendations. For both rAg and rPCR tests, a positive result was considered to be a true positive. In primary care clinics, confirming a negative rAg result with a laboratory based molecular test was encouraged, but not mandated.

### Data collection

We included cases using the following criteria: (1) encounter location was an Intermountain urgent care clinic or primary care clinic, (2) patient underwent POC SARS-CoV-2 testing during the corresponding clinical visit with either rPCR or rAg, (3) cases had no prior record of testing positive for at least three months prior to the initial test, (4) patients were at least 18 years of age. Patients who tested positive again after a duration of more than three months from their previous positive test were considered to have a new infection, and they were included in the study. A POC result was considered index positive if a patient tested positive at a clinic visit without any positive SARS-CoV-1 tests performed in the 90 days prior to the visit. Patients who initially tested negative at the index visit and subsequently tested positive within seven days of the visit by any test modality was classified as a false negative. We did not determine a true false negative rate as repeat testing following a negative index test was infrequently performed. We excluded patients who were tested for preprocedural purposes, and individuals who had been tested for SARS-CoV-2 within a month of the study’s commencement. This step was taken to ensure that subsequent visits were not misconstrued as initial encounters. We collected demographic information, underlying medical comorbidities, COVID-19 risk severity score (a validated prediction tool that integrates age, comorbidities, and ethnicity to predict risk of hospitalization and mortality),^6^ and vaccination status (either 0 vaccines, 1–2 vaccines, or 3+ vaccines at initial visit). We collected antibiotic and COVID-specific antiviral data administered within 24 hours of an index clinic visit.

### Statistical analysis

To investigate the association of individual characteristics, underlying medical conditions, vaccination status, and test modality with each outcome, we performed univariate logistic regression and calculated odds ratios (OR) and 95% confidence intervals (CI) for each outcome. Outcomes of interest included: treatment with antiviral agent, treatment with an antibiotic typically used for pneumonia (Supplemental Table 1) within 24 hours of index visit. We then analyzed multivariate logistic regression models for each outcome by sex, risk score, race, and vaccination status. To eliminate the influence of confounding factors based on clinical setting to the greatest extent possible, we performed 3:1 propensity score matching (PSM) for the rPCR and rAg groups with random matching and an absolute difference of 0.01 between propensity scores. We matched groups by age, gender, ethnicity, COVID-19 severity risk score, and underlying co-morbidities. We conducted statistical analyses using R version 4.3.2 and SAS 9.4 (SAS Institute Inc., Cary, NC, USA).

## Results

### Unmatched analysis

During the study period 104,364 patients underwent testing with a rapid test in the ambulatory setting (93,128 by rPCR and 11,236 by rAg). Of these, the percent positive was higher overall with rPCR compared to rAG (26.2% vs 20.8%; *P* < .0001) with more patients testing positive at the initial visit with rPCR compared to rAg (98.5% vs 94.1%, *P* < .0001). While confirming a negative rAg with PCR was encouraged, only 1,300 (14.7%) were submitted for one or more SARS-CoV-2 tests within 7 days of the index negative rAg test and 124 (5.9%) tested positive. Re-testing was infrequent following a negative rPCR (2.8%) with fewer false negative results (1.5%).

We observed significant differences in patient characteristics (Table 1) among patients seen in urgent care clinics compared to those seen in primary care clinics. Patients seen in urgent care clinics, where rPCR testing was used, were significantly younger, less likely to be vaccinated, and had fewer comorbidities than those tested in primary care clinics. Overall, 24% of patients received COVID-19 antiviral prescription (38% in primary care clinics and 22% in urgent care clinics, *P* < .001). Antibiotic use was infrequent with 10.8% of SARS-CoV-2 positive patients receiving antibiotics within 24 hours of the index clinic visit (13.4% in primary care clinics vs 10.1% in urgent care clinics, *P* < .001). When broken down by antibiotics typically used for pneumonia, 11.4% of the rAg cohort and 8.3% of the rPCR cohort received antibiotics (*P* < .001).

**Table 1.** Demographics and comorbidities of ambulatory participants with COVID-19

	Overall, N = 24,133	rAg, N = 2,152^1^	rPCR, N = 21,981^1^	*P*-value^2^
Age	46 (19)	54 (19)	46 (19)	<.001
Gender				.5
Male	10,203 (42%)	931 (43%)	9,272 (42%)	
Female	13,928 (58%)	1,221 (57%)	12,707 (58%)	
Trans-gender	2 (<.1%)	0 (0%)	2 (<0.1%)	
COVID-19 Vaccination status; n				<.001
No vaccination	8,015 (33%)	576 (27%)	7,439 (34%)	
1–2 doses	7,496 (31%)	633 (29%)	6,863 (31%)	
3+ doses	8,622 (36%)	943 (44%)	7,679 (35%)	
COVID severity risk score	5.3 (3.1)	6.7 (3.4)	5.2 (3.0)	<.001
Immunosuppression	323 (1.3%)	45 (2.1%)	278 (1.3%)	.001
Diabetes mellitus	2,992 (12%)	469 (22%)	2,523 (11%)	<.001
Metastatic cancer	256 (1.1%)	39 (1.8%)	217 (1.0%)	<.001
Chronic pulmonary disease	8,242 (34%)	956 (44%)	7,286 (33%)	<.001
Chronic kidney disease	1,322 (5.5%)	241 (11%)	1,081 (4.9%)	<.001
Liver disease	2,459 (10%)	335 (16%)	2,124 (9.7%)	<.001
Obesity	5,848 (24%)	747 (35%)	5,101 (23%)	<.001
Congestive heart failure	1,038 (4.3%)	182 (8.5%)	856 (3.9%)	<.001
Cardiac arrhythmia	4,859 (20%)	571 (27%)	4,288 (20%)	<.001
Neurologic disorder	1,671 (6.9%)	208 (9.7%)	1,463 (6.7%)	<.001
Hypertension	6,677 (28%)	992 (46%)	5,685 (26%)	<.001
History of stroke	1,266 (5.2%)	196 (9.1%)	1,070 (4.9%)	<.001
Race and ethnicity:				
White	21,165 (88%)	1,928 (90%)	19,237 (88%)	.005
Hispanic	3,660 (15%)	337 (16%)	3,323 (15%)	.5
Asian	659 (2.7%)	43 (2.0%)	616 (2.8%)	.029
Black or African American	349 (1.4%)	18 (0.8%)	331 (1.5%)	.013
Native Hawaiian or Pacific Islander	355 (1.5%)	18 (0.8%)	337 (1.5%)	.010
American Indian or Alaskan Native	218 (0.9%)	25 (1.2%)	193 (0.9%)	.2
Miscellaneous	1,387 (5.7%)	120 (5.6%)	1,267 (5.8%)	.7
Communities of color	5,774 (24%)	485 (23%)	5,289 (24%)	.11
Received respiratory antibiotic prescription	2,063 (8.5%)	245 (11.4%)	1,818 (8.3%)	<.001
Received antiviral prescription	5,712 (24%)	811 (38%)	4,901 (22%)	<.001
Received both respiratory antibiotic and antiviral prescription	446 (1.8%)	81 (3.8%)	365 (1.7%)	<.001
Emergency room visit within 30 days of initial encounter	1,134 (4.7%)	110 (5.1%)	1,024 (4.7%)	.3
Hospitalization within 30 days of initial encounter	361 (1.5%)	45 (2.1%)	316 (1.4%)	.017
Death within 30 days of initial encounter	18 (<0.1%)	3 (0.1%)	15 (<0.1%)	.2

1 Mean (SD); n (%).

2 Wilcoxon rank sum test; Pearson’s Chi-squared test; Fisher’s exact test.

On univariate logistic regression analysis of the entire study population, each comorbidity was associated with a significantly increased odds of receiving antibiotics; immunosuppression (Odds ratio (OR):1.6 [1.2–2.2], *P* = .004) and congestive heart failure (OR: 1.6 [1.3–1.9], *P* < .001) were associated with the highest odds (Table 2). White non-Hispanic patients were significantly more likely to receive antibiotics typically used for pneumonia (OR: 1.4 [1.2–1.6], *P* < .001). When covariables were analyzed in a multivariate model, chronic lung disease (OR 1.4 [1.2–1.5]) and non-Hispanic white race (OR 1.3 [1.1–1.5]) were associated with receiving an antibiotic whereas patients tested with PCR and who received one or more doses of the COVID vaccine were less likely to have received antibiotics (Table 2).

**Table 2.** Odds ratios and 95% confidence intervals for univariate and multivariate logistic regression models: COVID-19 patients receiving antibiotics for pneumonia by demographics, comorbidities, vaccination status, and rapid diagnostics

	Univariate logistic regression		Multivariate logistic regression	
	Odds ratios (95% confidence intervals)	*P*-value	Odds ratios (95% confidence intervals)	*P*-value
Age	1.0 (1.0–1.01)	<.001	1.0 (1–1.01)	<.001
Immunosuppression	1.6 (1.1–2.2)	.004	1.7 (.8–3.1)	.2
Diabetes	1.2 (1.1–1.4)	.003	.9 (.8–1.0)	.09
Metastatic carcinoma	1.5 (1–2.2)	.02	.7 (.3–1.6)	.4
Chronic lung disease	1.5 (1.0–1.7)	<.001	1.4 (1.2–1.5)	<.001
Renal disease	1.5 (1.3–1.8)	<.001	1.1 (.9–1.3)	.4
Liver disease	1.4 (1.2–1.6)	<.001	1.2 (.9–1.3)	.06
Obesity	1.3 (1.2–1.4)	<.001	1.1 (.9–1.2)	.2
History of stroke	1.4 (1.2–1.7)	<.001	1.0 (.8–1.2)	.9
Hypertension	1.4 (1.3–1.6)	<.001	1.1 (.9–1.2)	.5
Neurologic disease	1.3 (1.1–1.5)	.002	1.0 (.9–1.2)	.7
Congestive heart failure	1.6 (1.3–1.9)	<.001	1.1 (.9–1.4)	.3
Cardiac arrhythmia	1.3 (1.2–1.4)	<.001	1.0 (.9–1.2)	.7
Total comorbidities	1.1 (1.1–1.1)	<.001		
Risk score	1.1 (1.0–1.1)	<.001		
Gender, male	1.0 (.9–1.1)	.8		
Race and ethnicity:				
Black/African American	.6 (.4–1.0)	.06		
Native Hawaiian/Pacific Islander	.8 (.5–1.2)	.3		
White	1.4 (1.2–1.6)	<.001	1.3 (1.1–1.5)	.002
Miscellaneous	.8 (.7–1.0)	.1		
Ethnic Hispanic	.8 (.7–.9)	.001	.9 (.8–1)	.06
Vaccination Status (1–2)	.8 (.8–.9)	.003	.8 (.7–.9)	<.001
Vaccination Status (3+)	.9 (.8–1.0)	.128	.7 (.7–.8)	<.001
Rapid PCR diagnostic	.7 (.6–.8)	<.001	.8 (.7–.9)	.001

### Matched analysis

Due to significant imbalances in baseline characteristics between patients tested in urgent care compared to primary care clinics, we performed regression analysis on a propensity matched cohort to further explore whether POC test modality was associated with antibiotic use and COVID-19 antiviral use. Our propensity matched cohort included 8,675 SARS-CoV-2 positive cases of which 6,523 were tested with rPCR and 2,152 were tested with rAg. Propensity matching was successful in eliminating differences in demographics, COVID-19 severity risk score, and comorbid conditions (Supplemental Table 2). Supplemental Figure 1 shows a covariate balance plot for the pre and postpropensity score matched groups. A greater proportion of patients tested with rAg received COVID-19 antiviral therapy (37.7% vs 33.7%, *P* < .001) and antibiotics typically used for pneumonia (11.4% vs 8.5%, *P* < .001).

On univariate logistic regression, several comorbidities were significantly associated with receiving antibiotics within 24 hours of index visit: chronic lung disease (Odds ratio (OR): 1.5 [1.3–1.8], *P* < .001), chronic kidney disease (OR: 1.4 [1.1–1.8], *P* = .02), liver disease (OR: 1.3 [1.0–1.6], *P* = .04), obesity (OR: 1.3 [1.1–1.5], *P* = .006), hypertension (OR: 1.3 [1.1–1.6], *P* = .002), and congestive heart failure (CHF) (OR: 1.5 [1.1–1.9], *P* = .008) (Table 3). White non-Hispanic patients were more likely to receive antibiotics typically used for pneumonia (OR 1.6 [1.2–2.2], *P* = .006) and antiviral therapy (OR: 1.3, *P* < .001) compared to patients of other races and ethnicities (Table 3). In our multivariate model, all predictors with a *P* value of less than. 05 on univariate analysis (aside from COVID-19 risk severity score as it was a composite predictor and communities of color as it is the reciprocal of white non-Hispanic) were included. Patients tested by rPCR remained less likely to receive antibiotics (OR: 0.8 [0.7–1.0], *P* = .03) (Table 3).

**Table 3.** Odds ratios and 95% confidence intervals for univariate and multivariate logistic regression models: COVID-19 patients receiving antibiotics for pneumonia by demographics, comorbidities, vaccination status, and rapid diagnostics for unmatched cohort

	Univariate logistic regression		Multivariate logistic regression	
	Odds ratios (95% confidence intervals)	*P*-value	Odds ratios (95% confidence intervals)	*P*-value
Age	1.01 (1–1.01)	.005	1.0 (1.0–1.0)	.433
Immunosuppression	1.5 (.9–2.5)	.1		
Diabetes	1.1 (.9–1.4)	.2		
Metastatic carcinoma	1.5 (.8–2.5)	.2		
Chronic lung disease	1.5 (1.3–1.8)	<.001	1.4 (1.2–1.7)	<.001
Renal disease	1.4 (1.1–1.8)	.02	1.1 (.8–1.5)	.5
Liver disease	1.3 (1.0–1.6)	.04	1.1 (.8–1.3)	.6
Obesity	1.3 (1.1–1.5)	.006	1.2 (1.0–1.4)	.1
History of stroke	1.2 (.9–1.5)	.3		
Hypertension	1.3 (1.1–1.6)	.002	1.04 (.8–1.3)	.7
Neurologic disease	1.0 (.8–1.4)	.8		
CHF	1.5 (1.1–1.9)	.008	1.1 (.8–1.5)	.4
Cardiac arrhythmia	1.2 (1.0–1.4)	.2		
Total comorbidities	1.1 (1.0–1.1)	<.001		
Risk score	1.1 (1.0–1.1)	<.001		
Gender, male	.9 (.7–1.0)	.08		
Race and ethnicity:				
Black or African American	.8 (.3–1.7)	.7		
Native Hawaiian or Pacific Islander	.4 (.1–1.1)	.1		
White	1.6 (1.2–2.2)	.006	1.5 (1.1–2.1)	.01
Miscellaneous	.7 (.4–1.0)	.09		
Hispanic	.8 (.6–1.0)	.1		
Communities of color	.7 (.6–.9)	.003		
Vaccination Status (1–2)	.9 (.7–1.2)	.4		
Vaccination Status (3+)	.9 (.8–1.1)	.5		
Rapid PCR diagnostic	.8 (.7–1.0)	.02	.8 (.7–1.0)	.03

### Discussion

Our study demonstrates significant impact of POC testing for COVID-19 in an ambulatory setting, particularly highlighting the differences between rPCR and rAg testing. While both rapid methods led to rapid diagnosis at the point of care, we found that rPCR had better performance with fewer false negative results. We could not determine the true false negative rate for rAg because providers infrequently obtained RT-PCR confirmation of a negative rAg result, despite auto-order generation. However, prior to wide-spread availability of rPCR tests in our system, we used rAg test in our ERs with negative or invalid results receiving PCR confirmation. Over a one-month period, 99.1% of the negative rAg were tested by PCR with a false negative rate of 30.8% (unpublished data). Other studies have shown that molecular methods have greater analytical sensitivity compared to antigen-based testing.^7,8^

A key finding in our study is the difference in antibiotic prescribing patterns between the two testing modalities. Overall, 10.8% of adults who tested positive for SARS-CoV-2 were prescribed antibiotics, surpassing earlier reports of less than 4%.^2,3^ However, this rate is much lower than the 30% reported by Tsay et al. for adults 65 years and older earlier in the pandemic, when POC testing was not widely available.^4^ A prior study quantifying antibiotic use for respiratory conditions in Intermountain Health urgent care clinics demonstrated that antibiotics were administered in 47.8% of encounters and declined to 33.3% after multimodal intervention.^9^ Therefore, our results suggests that POC testing for SARS-CoV-2 likely contributes to improved antibiotic use in the ambulatory setting. There is limited data regarding the rate of bacterial co-infection in ambulatory patients with COVID-19, but it is likely lower than the estimated 4–5% of co-infections reported for hospitalized patients with COVID-19.^10,11^

In our regression analysis of the unmatched cohort and propensity matched cohort, we found that antibiotic prescribing was less likely if testing was with rPCR. Patients with underlying chronic pulmonary disease were more likely to have received antibiotics after testing positive for SARS-CoV-2 as observed in analysis of both total and propensity matched cohorts. This is not surprising given poor pulmonary reserve and higher risk of progressing to severe disease. We also found that non-Hispanic white patients were more likely to be treated with antibiotics. Similar trends between race/ethnicity and antibiotic prescribing was observed in our urgent care clinics preCOVID-19 in earlier studies^12^ and our results align with other studies showing non-Hispanic white patients receive significantly more antibiotics for COVID-19 in the ambulatory setting than other racial and ethnic groups.^3,4^ Studies prior to the COVID-19 pandemic also demonstrated increased antibiotic prescription for non-Hispanic white children,^13^ although other studies have demonstrated antibiotic overuse was more prevalent for black and Hispanic patients.^14,15^ The causes of ethnic disparities in antibiotic have yet to be identified.

The method used for point of care testing in our health system was not random and was determined by the care setting. We assumed *a priori* that the patient population would be similar between urgent care and primary care settings, however discovered that patients seen in primary care clinics where rAg testing was performed had significantly greater underlying comorbidities. Differences in antibiotic prescribing based on test method may reflect underlying differences in clinical practice settings between urgent care and primary care clinics. Prior studies have demonstrated that practice setting can significantly influence rates of antibiotic prescription for COVID-19 and other respiratory infections.^4,15,16^ To address potential confounding, we employed propensity score matching. Multivariate analysis of the well-balanced propensity cohort revealed that differences in antibiotic use by test method persisted, with primary care clinicians more likely than their urgent care counterparts to prescribe both antibiotics. There are several factors to consider which may explain these differences. First, patient satisfaction has been shown to increase when antibiotics are prescribed for respiratory tract infections, potentially influencing clinicians to prescribe in order to maintain a patient-physician relationship.^17–19^ Second, just prior to the study period, targeted antimicrobial stewardship initiatives were implemented in Intermountain Health urgent care clinics but had not yet been extended to primary care clinics.^20^ Finally, the type of diagnostic test and the clinician’s confidence in that test may influence the decision to prescribe antibiotics.

Similarly, primary care clinicians were more likely to prescribe antiviral therapy. The most likely explanation is that patients seen in the primary care clinics had significantly greater comorbidities, which was better balanced after propensity matching (Supplemental Table 2). During this phase of the pandemic, all patients who tested positive for COVID in our system were assigned a risk score.^6^ Patients deemed at high risk for severe disease were subsequently recommended for antiviral therapy. After adjusting for comorbidities, the difference in antiviral prescriptions between primary care and urgent care clinics declined from 16% to 4%. Another possible contributing factor is a better understanding of medication list by primary care clinicians compared to urgent care clinicians. Given the drug-drug interactions with antiviral medications-such as nirmatrelvir/ritonavir—clinicians with incomplete medication histories may be more hesitant to prescribe antiviral therapy.^21^

This study has several limitations. First, we did not evaluate baseline outpatient antibiotic prescriptions early in the pandemic when POC tests were not yet available and diagnostic testing for SARS-CoV-2 was performed in hospital-based laboratories. Second rPCR and rAg were used in different ambulatory settings (urgent care vs primary care) with significant differences in patient characteristics that could influence antibiotic use. We attempted to control for these differences by analyzing a propensity matched cohort. Finally, our analysis conditioned on SARS-CoV-2 positive cases, which allowed us to assess prescribing after a confirmed diagnosis. However, this approach does not emulate an intention-to-treat framework and instead reflects a mechanistic effect (eg, antibiotic avoidance once COVID-19 is confirmed). Future studies using intention-to-treat designs could better capture the overall impact of rAg versus rPCR testing. Despite these limitations, our study included a large, diverse sample size with a standardized approach to patient care in each ambulatory setting. These strengths, along with well-balanced propensity-score matched groups, allowed us to evaluate the impact of different testing modalities and different ambulatory clinical settings on the management of COVID-19.

In conclusion, we show low rates of antibiotic use in ambulatory settings where POC testing for SARS-CoV-2 was deployed. Molecular testing with rPCR outperformed antigen testing in diagnosing COVID-19 and was associated with the lowest likelihood of antibiotic use.

## Supporting information

10.1017/ash.2026.10362.sm001 Williams et al. supplementary material Williams et al. supplementary material

## Supplementary material

The supplementary material for this article can be found at https://doi.org/10.1017/ash.2026.10362.
