# Supplementary material for: Real-world impact of point-of-care testing for SARS-CoV-2 in an ambulatory setting of an integrated health network
Source: Antimicrob Steward Healthc Epidemiol. 2026 Apr 24;6(1):e120. doi: 10.1017/ash.2026.10362 (PMC13126208; doi:10.1017/ash.2026.10362)
Supplement: Williams et al. supplementary material [file S2732494X26103623sup001.docx]

| Supplemental Table 1. Antibiotics used for pneumonia |
| --- |
| Amoxicillin  Amoxicillin-Clavulanate  Azithromycin  Ceftriaxone  Cefdinir  Cefixime  Cefpodoxime  Cefuroxime  Clarithromycin  Doxycycline  Levofloxacin  Moxifloxacin |

| Supplemental Table 2. Demographics and comorbidities for propensity score matched groups based on the type of rapid diagnostic. | | | |
| --- | --- | --- | --- |
| Characteristic | **rAg**, N = 2,152*^1^* | **rPCR**, N = 6,523*^1^* | **p-value***^2^* |
| Age | 54 (19) | 53 (19) | 0.3 |
| Gender |  |  | 0.7 |
| Male | 931 (43%) | 2,787 (43%) |  |
| Female | 1,221 (57%) | 3,736 (57%) |  |
| COVID-19 Vaccination Status |  |  | 0.4 |
| No Vaccinations | 576 (27%) | 1,726 (26%) |  |
| 1-2 Vaccinations | 633 (29%) | 1,818 (28%) |  |
| 3+ Vaccinations | 943 (44%) | 2,979 (46%) |  |
| Risk Score | 6.7 (3.4) | 6.6 (3.4) | 0.4 |
| Immunosuppression | 45 (2.1%) | 126 (1.9%) | 0.6 |
| Diabetes Mellitus | 469 (22%) | 1,380 (21%) | 0.5 |
| Metastatic Cancer | 39 (1.8%) | 110 (1.7%) | 0.7 |
| Chronic Lung Disease | 956 (44%) | 2,863 (44%) | 0.7 |
| Renal Disease | 241 (11%) | 667 (10%) | 0.2 |
| Liver Disease | 335 (16%) | 1,006 (15%) | 0.9 |
| Obesity | 747 (35%) | 2,273 (35%) | 0.9 |
| History of Stroke | 196 (9.1%) | 586 (9.0%) | 0.9 |
| Hypertension | 992 (46%) | 2,924 (45%) | 0.3 |
| Neuro | 208 (9.7%) | 643 (9.9%) | 0.8 |
| CHF | 182 (8.5%) | 517 (7.9%) | 0.4 |
| Cardiac Arrythmia | 571 (27%) | 1,704 (26%) | 0.7 |
| Race and Ethnicity: | | | |
| White | 1,928 (90%) | 5,773 (89%) | 0.2 |
| Hispanic | 337 (16%) | 1,039 (16%) | 0.8 |
| Asian | 43 (2.0%) | 154 (2.4%) | 0.3 |
| American Indian/Alaskan Native | 25 (1.2%) | 53 (0.8%) | 0.14 |
| Black/African American | 18 (0.8%) | 92 (1.4%) | 0.039 |
| Native Hawaiian/Pacific Islander | 18 (0.8%) | 93 (1.4%) | 0.035 |
| Miscellaneous Race | 120 (5.6%) | 358 (5.5%) | 0.9 |
| Communities of Color | 485 (23%) | 1,534 (24%) | 0.4 |
|  | | | |
| Received Antibiotic Prescription | 289 (13%) | 689 (11%) | <0.001 |
| Received Respiratory Antibiotic Prescription | 245 (11%) | 557 (8.5%) | <0.001 |
| Received Antiviral Prescription | 811 (38%) | 2,197 (34%) | <0.001 |
| ^1^ Mean (SD); n (%) | | | |
| ^2^ Wilcoxon rank sum test; Pearson’s Chi-squared test; Fisher’s exact test | | | |


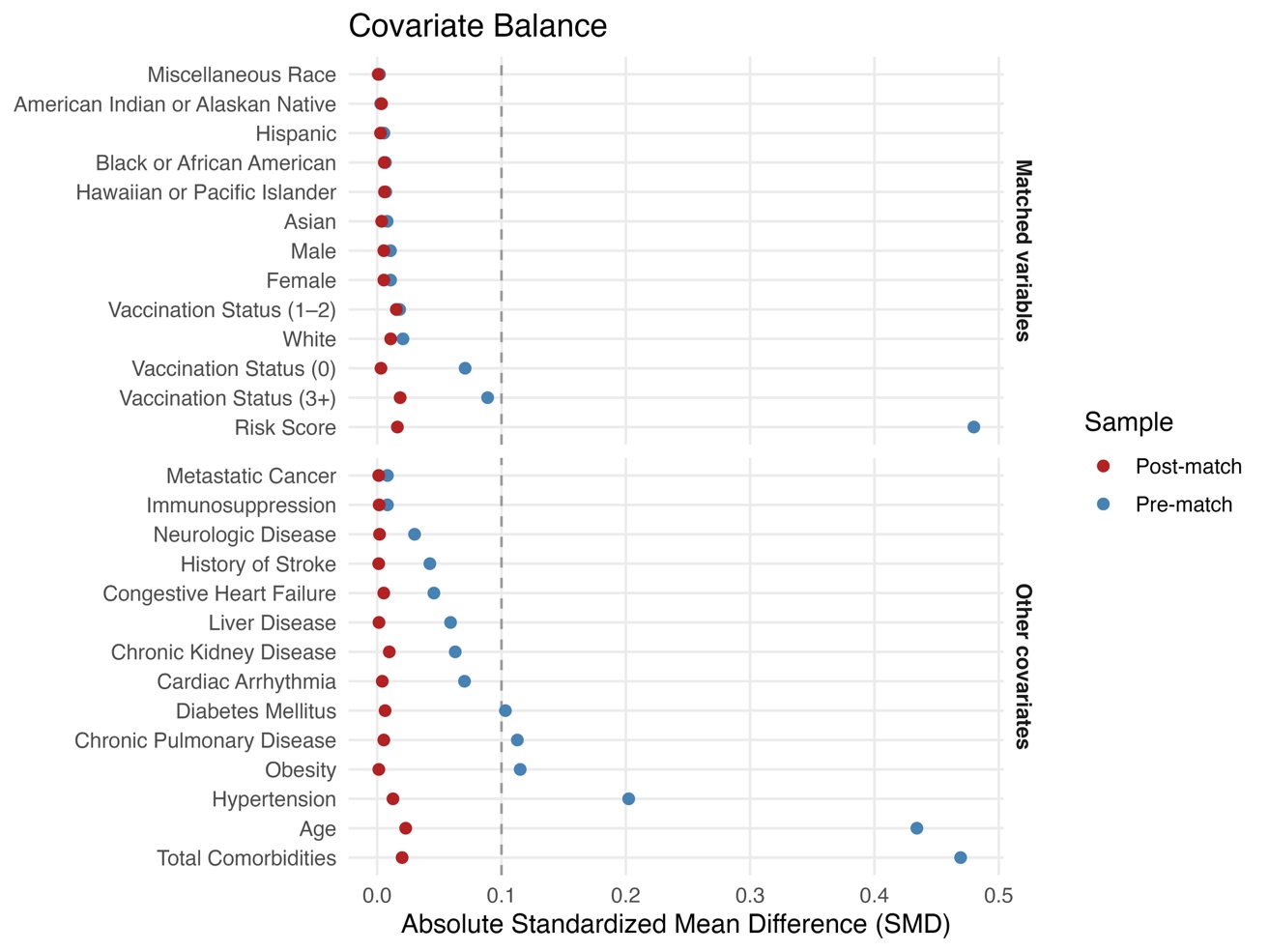


**Supplemental Figure 1.** Covariate Balance Plot for the pre- and post- propensity score matched groups. Points represent the absolute standardized mean difference for each covariate comparing rapid PCR (rPCR) and rapid antigen (rAg) cohorts. Covariates included in the propensity score model are listed above and other clinical variables of interest are listed below.
